# Hydrogel-Film-Fabricated Fluorescent Biosensors with Aggregation-Induced Emission for Albumin Detection through the Real-Time Modulation of a Vortex Fluidic Device

**DOI:** 10.3390/molecules28073244

**Published:** 2023-04-05

**Authors:** Qi Hu, Xuan Luo, Damian Tohl, Anh Tran Tam Pham, Colin Raston, Youhong Tang

**Affiliations:** 1 Australia-China Joint Research Centre on Personal Health Technologies, Medical Device Research Institute, Flinders University, Adelaide, SA 5042, Australia; qi.hu@flinders.edu.au (Q.H.); damian.tohl@flinders.edu.au (D.T.); anh.pham@flinders.edu.au (A.T.T.P.); 2 Institute for NanoScale Science and Technology, College of Science and Engineering, Flinders University, Adelaide, SA 5042, Australia; xuan.luo@flinders.edu.au (X.L.); colin.raston@flinders.edu.au (C.R.)

**Keywords:** aggregation-induced emission, fluorescence biosensor, human serum albumin, hydrogel films, vortex fluidic device

## Abstract

Hydrogels have various promising prospects as a successful platform for detecting biomarkers, and human serum albumin (HSA) is an important biomarker in the diagnosis of kidney diseases. However, the difficult-to-control passive diffusion kinetics of hydrogels is a major factor affecting detection performance. This study focuses on using hydrogels embedded with aggregation-induced emission (AIE) fluorescent probe TC426 to detect HSA in real time. The vortex fluidic device (VFD) technology is used as a rotation strategy to control the reaction kinetics and micromixing during measurement. The results show that the introduction of VFD could significantly accelerate its fluorescence response and effectively improve the diffusion coefficient, while VFD processing could regulate passive diffusion into active diffusion, offering a new method for future sensing research.

## 1. Introduction

Human serum albumin (HSA), as the most abundant plasma protein, (55–60%) [1], is a significant biomarker for clinical diagnosis, particularly on renal disease and proteinuria [2]. It also plays vital roles in many physiological functions, including the maintenance of normal plasma colloidal osmotic activity [3], the specific attractive force for the retention of positively charged solutes in blood vessels [4], and good scavenging ability towards free radicals [5]. Therefore, HSA detection is greatly important in clinical sensing due to its biological effects and medical benefits. Additionally, the α-helical domains of the HSA structure where Primary Binding Sites I and II located at Subdomains IIA and IIIA tend to react with larger heterocyclic compounds and dicarboxylic acid, and small molecules allow for numerous endogenous and exogenous substances to bind with HSA via varying ligand binding affinity [6,7,8]. The current methods for identifying albumin utilise its characterised affinity, and immunoassay and colorimetry are very common measurement methods [9]. Generally, immunoassays (limit of detection (LOD): 2–10 mg/L) are undesirable for real-time HSA detection due to being time-consuming, expensive, and complicated processes, and their low detection sensitivity and small detection window in colorimetry (LOD: 150 mg/L) hinder accurate detection [10,11,12].

Biosensors with aggregation-induced emission (AIE) [13,14,15,16] are one of the most effective tool for HSA detection due to their high sensitivity, rapid response, specific selectivity, operational simplicity, cost efficiency, and real-time and in vitro sensing [17,18,19,20]. Tu and co-workers reported a water-soluble luminogen with AIE characteristics, TPE–4TA, in their work on HSA detection in a buffer and human urine bioapplication [21] that had a wide dynamic range (0.02–3000 mg/L), a very low detection limit (0.21 nM), and strong binding affinity towards HSA (KD = 0.25 μM). In our previous research, we also demonstrated novel AIE-based fluorescent dye TC426 for detecting urinary albumin via selective site-specific binding [22]. TC426 is nonemissive in polar aqueous solution, but lights up after coming into contact with albumin, and hydrophobic interaction forces the AIE molecules to enter the interior domain of Sudlow Site I on secondary and tertiary structures of HSA, thereby enhancing fluorescent performance. Moreover, it exhibited a linear range of microalbumin (20–200 mg/L) with a low LOD of 3.74 nM in real application.

Hydrogels, as flexible three-dimensional materials, are widely utilised due to their nontoxicity, low cost, easily customisable structure and volume/shape variations, and good biocompatibility [23,24,25]. The functional structure of their cross-linked hydrophilic network and high water content can be manipulated for potential applications such as biomarker detection [26], drug delivery [27], and shape support [28]. Research has focused on applying hydrogels to biomedical fields. Sun and his colleagues fabricated calcium alginate–polyacrylamide (CA–PAM) hybrid hydrogels with good mechanical characteristics through the combination of covalently cross-linked acrylamide (AM) and ionically cross-linked sodium alginate (SA) [29]. This hydrogel had a fractural energy of roughly 9000 Jm^−2^ and could be stretched more than 20 times its original length even though it had about 90% water content. The covalently crosslinked network’s crack-bridging capability and the ionic crosslinked network’s hysteresis contributed to the hydrogel’s toughness as a whole. The adhesive properties of such hydrogels allow for hydrophobic substances to be fixed stably inside through electrostatic interactions, covalent bonds, or physical interpenetration. On the other hand, carrageenan is a class of natural polymers that are widely used in the pharmaceutical and food industries as gelling agents, emulsifiers, thickeners, or stabilisers [30]. Mihaila et al. developed the kappa carrageenan via physical and chemical cross linking for the demands of tissue engineering [31]. By adjusting the degree of methacrylate and fabricating through the micromolding method, kappa carrageenan exhibited good hydration, dissolution profile, morphology, and mechanical and rheological properties, and had a spatially controlled geometry and cell distribution patterns. The results showed that the combination of its chemical and physical cross-linking procedures enabled the formation of hydrogels with highly versatile physical and chemical properties while maintaining the viability of encapsulated cells. On the basis of this evidence, these impressive hydrogel materials have potential to serve as a matrix for the construction of fluorescent hydrogel films.

A current research branch focuses on introducing AIE units into polymeric hydrogels to enhance the fluorescent properties through their surface adsorption, hydrophobic interaction, self-assembly, hydrogen bonding, or other effects [32,33]. Luminogen-based hydrogel films are final products that have the advantages of both AIE biosensors and hydrogels. The majority of AIEgens belong to hydrophobic organic molecules with nonplanar propeller configurations that hardly fluoresce in their normal form, but emit bright light when aggregated. The hydrophilic hydrogel matrix where the spontaneous aggregation of AIEgens inhibits their intramolecular movements could further amplify fluorescence [34]. Furthermore, the encapsulation of an AIE-based probe within a hydrophilic cross-linked structure is crucial for improving the environmental friendliness and biocompatibility of AIEgens in biomarker detection [35]. However, some drawbacks of hydrogel films that may have impact detection also need attention. Specifically, the sealed construction of hydrogel forces the luminogens to react with the biomarker from outside to inside, thereby prolonging the detection time [36]. Additionally, the high swelling behaviour may promote light scattering and increase the internal structural clearance of hydrogels, resulting in weakened signals and reduced detection sensitivity, while its diffusion kinetics is predominantly passive and difficult to control [37]. Improving these unfavourable factors is a vital first step in improving the performance of fluorescent hydrogels.

**Figure 1:**
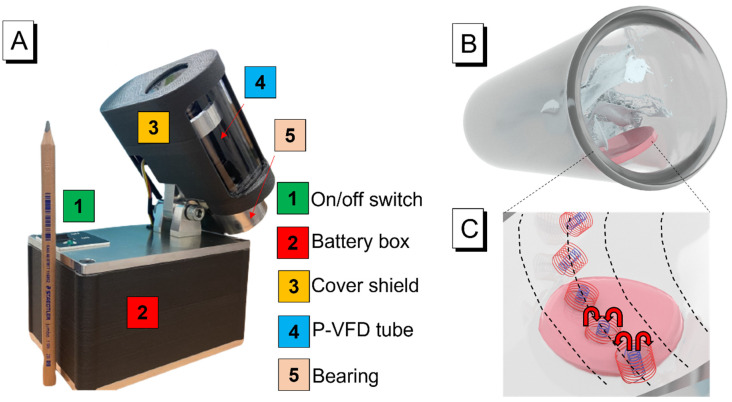
(**A**) Portable VFD used in this study; (**B**) interaction process between the solution and hydrogel film adhered to the tube wall; (**C**) efficient spinning top flow-mediated transfer of reactants into and out of the hydrogel matrix.

The vortex fluidic device (VFD, Figure 1A) is a rotational system and liquid thin-film processing platform that was intensively used for applications such as material synthesis [38], micromixing [39], chemical reactions [40], and improving mechanical properties [41]. The mechanical energy generated by a VFD could be effectively applied to reactions to precisely control physical/chemical progress [42]. Accelerating the reaction kinetics, modifying the chemical reactivity, and enabling fast fluidic exchange are all significant effects of centrifugal or shear pressure from VFDs [43,44]. More specifically, this microfluidic platform allows for a continuous flow operation with small sample volumes (≤1 mL) with adjustable angle, rotational speed, and steering to produce dynamic thin films on the tube-wall surface and apply shearing stress during dynamic mixing. The liquid thin film offers many advantages, including a large surface area, high shear rates, rapid heat and mass transfer, micromixing, and fluid pressure waves [45]. There have been several cases of hydrogel fabrication and optimisation using VFD. Recently, Tavakoli et al. produced the tuning surface morphology of fluorescent hydrogels using a VFD [46]. Their results showed that the physically cross-linked hydrogels fabricated with the VFD technology had excellent fluorescence and self-adhesion, and adjustable morphologies. VFDs enable the intense micromixing of various ingredients, resulting in improved spatial distribution and more homogeneous end products. Luo et al. also reported silica hydrogels with embedded laccase nanoflowers (LNFs) for real-time biosensing under vortex-fluidic-mediated fabrication [47]. VFDs significantly reduce the LNF production rate and further enhance LNF activity. Additionally, LNF@silica-coated VFD tubes could be used to track enzymatic processes, leading to a dramatic rise in catalytic activity (up to 16-fold).

Given the above-mentioned characteristics of VFD, this study uses the VFD technology to accelerate intra- and extrafilm hydrodynamics in real time while enabling the AIEgens to be uniformly micromixed under shearing force, thus reducing the detection time, enhancing the fluorescence signal, and forming an active diffusion-controlled system. In this work, two hydrogel matrices, acrylamide–alginate (AAm–Alg) and carrageenan, were employed in conjunction with an AIEgen, TC426 [22], to detect HSA in real time and investigate their characterisations under VFD regulation. The introduction of VFD technology could meet the higher requirements of biomarker detection in fluorescent hydrogels.

## 2. Results and Discussion

### 2.1. Kinetics of Fluorescent Response and VFD Modulation

**Figure 2:**
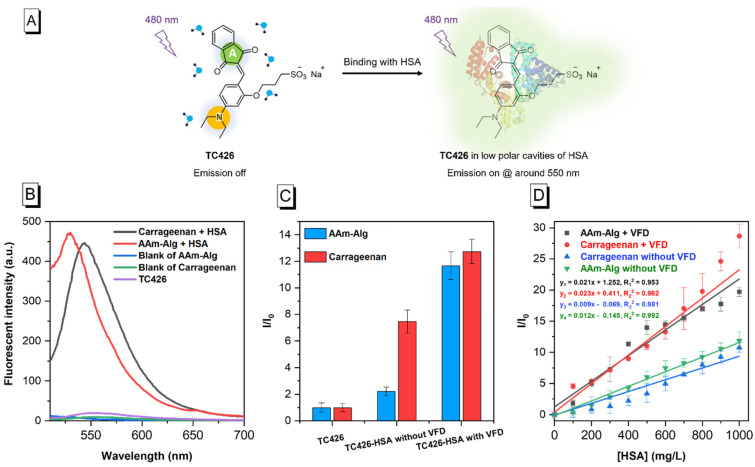
Characterisation of hydrogel films using TC426. (**A**) Schematic of the working mechanism of TC426 in HSA detection [12]; (**B**) FL spectra of TC426 in two matrices: AAm−Alg and carrageenan under different conditions; (**C**) effect of TC426-based hydrogel films with and without VFD modulation; (**D**) standard curve of TC426-embedded hydrogel films under different HSA concentrations ranging from 0 to 1000 mg/L with/without VFD processing. TC426 = 10 μM, λ_ex_ = 480 nm, I_0_ = intensity of HSA = 0 mg/L; VFD was used for 2 and 4 min in AAm–Alg + TC426 and Carrageenan + TC426, respectively.

TC426 exhibited typical AIE characteristics in the hydrogel films (Figure 2A). Figure 2B shows that TC426 maintained a nonfluorescent state in both matrices: acrylamide–alginate and carrageenan. However, the introduction of HSA triggered green fluorescence that immediately produced an emission at the peaks of 530 and 545 nm. When HSA variation was in the range from 0 to 1000 mg/L, the ligand bonding of TC426–HSA remained concentration-dependent while also indicating positive proportional intensity in both matrices (Figure S3). Additionally, a discrepancy of no more than 5.39% existed between the fluorescent intensity ranges presented with AAm–Alg and carrageenan.

On this basis, we further introduced a portable vortex fluidic device to observe the influence of hydrogel films on photoluminescence behaviours in real time. In order to quantify the changes during time-varying rotation, multiple aspects, including colour shift, fluorescent intensity, swelling ratio, dynamic loss, and temporal stability as corresponding characterisations depict whether the AIE properties of the hydrogels were significant in the comparison between the normal condition and that with VFD support. First, the colour gradient progressively became a more pronounced indicator (Figures S4 and S5). The hydrogels containing TC426 initially appeared orange-red in AAm–Alg and carrageenan. The high concentration of HSA (2000 mg/L) in the external surroundings began to progressively permeate into the interior structure of the hydrogels over time due to the concentration differential between the inside and outside of the film, and water absorption caused swelling. During this process, the chance of albumin binding to TC426 was significantly boosted, resulting in an increase in the number and size of TC426–HSA aggregates. The Mie effect was triggered by size changes and is reflected in the colour transformation from orange-red into pink, which was also consistent with the fluorescence spectrum. Colour change in AAm–Alg occurred at around 8 min in normal soaking (Figure S4A), but was advanced to the 2 min node under VFD support (Figure S4B). Similarly, a pink shift could be captured at 9 min without VFD (Figure S5A), and this phenomenon was accelerated for 5 min with a VFD (Figure S5B) in carrageenan. In this case, the time acceleration rates caused by the VFD were 60% and 50%.

Afterwards, the real-time variations for VFD processing were also recorded, as shown in Figure S6. The fluorescent signal presented a stable rise and peaked in the normal AAm–Alg soaking test at around 10 min, 8.47 times the beginning level (Figure S6A). The difference in the same conditions was that the intervention of VFD accelerated the fluid exchange, reaching the maximum (11.67 times the initial samples) at 2 min earlier (Figure S6B). Following that, the shearing force from the VFD progressively detached TC426 molecules from the film surface, and they turned into a solution that accounted for the weakening of the fluorescence signal between 2 and 10 min. Similarly, the absorption and natural flow of water enabled the TC426 probe in carrageenan to relatively slowly bind with albumin until it had reached a 11.83-fold increase at 10 min (Figure S6C). Nevertheless, the same situation took only 4 min to complete under VFD processing (12.32-fold), and intensities remained in steady state from 4 to 10 min; lastly, they became as strong as 14.30-fold the starting value (Figure S6D). Regarding the dynamic performance of swelling during normal soaking and VFD processing, the swelling ratio (Equation (1)) of AAm–Alg–TC426 rose steadily from 0 to 110.05% in 10 min, in accordance with water absorption (Figure S6A). However, VFD altered this circumstance. To be more precise, the swelling ratio of AAm–Alg–TC426 immediately reached 79.37% in the first 2 minutes of rotation and climbed slowly during the following 8 minutes, only rising by a total of 34.29% (Figure S6B). Different from AAm–Alg–TC426, carrageenan–TC426 demonstrated a similar kinetic tendency and range, and both soaking (Figure S6C) and VFD rotation (Figure S6D) were divided into two distinct trajectories: the rapid ascent phase (0–2 min) and the comparatively steady phase (2–10 min). At the first stage, they quickly rose to 43.45% and 50.26% respectively, and reached a plateau before rising to 62.55% and 63.94%, respectively.

Notably, a measurable decline in the fluorescent signal of AAm–Alg was observed in the previous VFD modulation (Figure S6B). The rotation of VFD unavoidably forced HSA into the hydrogel while transferring TC426 on the film surface into the external solution. Consequently, the determination of dynamic AIE loss is an essential step in realistically detecting quantitative biomarkers, optimising experimental outcomes, and comprehending the underlying fundamentals. Using Equation (5), we found the dynamic loss from 0 to 10 min during VFD for two different hydrogel basements (Figure S7A). The loss rate in AAm–Alg rapidly increased from 0 to 10.98% in the first 2 min and then steadily increased to 22.0% in the remaining 8 min. Moreover, the carrageenan film sharply increased by 5% within 1 min and eventually reached 13.3% at 10 min. Under the same experimental conditions, the loss ratio of carrageenan after 2 minutes was about half that of AAm–Alg, and its value was stable at around 10%. Moreover, the temporal stability of the fluorescent hydrogel film was assessed assuming that the dynamic loss was fully considered by modifying the rotational VFD time, as illustrated in Figure S7B. The fluorescence responses of the two matrices still retained 92.32% (AAm–Alg) and 95.84% (carrageenan) of their maximal peaks after 3 h.

The findings show that the intensity change of hydrogels was positively correlated with its colour shift. The orange-red of TC426 was contributed by the sodium sulfonate group in its chemical structure, and a pink transition occurred when the albumin macromolecules met the fluorescent dye as a result of the AIE molecules moving towards the Sudlow sites of albumin and the coaggregate size expanding. With the development of water diffusion, the fluorescence enhancement phenomena also became increasingly clear. Although the bidirectional exchange of the HSA solution and hydrogels showed that the dynamic loss and the dynamic binding occurred simultaneously, the high level of temporal stability proved that an outward–inward flow was the dominant motion. Comparing the two different hydrogels, the overall fluorescence enhancement performance of carrageenan was better than that of AAm–Alg, and the corresponding FL enhancing efficiency of carrageenan was 39.66% (in normal soaking) and 5.57% (using VFD) higher than that of AAm–Alg. Correspondingly, AAm–Alg–TC426 was eventually stabilised at a swelling ratio of around 110%, and its increase was approximately 1.8 times that of carrageenan–TC426 (around 60%).

Lastly, Figure 2C shows that the utilisation of VFD technology boosted the fluorescence response by 5.27-fold on AAm–Alg and 1.71-fold on carrageenan compared to their original level. Furthermore, the dynamic ranges of both hydrogels were linearly distributed with/without VFD (Figure 2D, R_1_^2^ = 0.953, R_2_^2^ = 0.962, R_3_^2^ = 0.981, R_4_^2^ = 0.992). The limits of detection (LOD, Equation (6)) for AAm–Alg and carrageenan without VFD intervention were 74.77 and 53.87 mg/L, respectively, while VFD further lowered these thresholds to 61.19 and 30.87 mg/L under the same conditions. The detection sensitivity of the standard curves under VFD control was 1.75 (AAm–Alg) and 2.56 (carrageenan) times higher than that of the non-VFD modulation.

### 2.2. Potential Mechanism

#### 2.2.1. Diffusion Characterisation 

The above investigations show that the swelling ratios of AAm–Alg and carrageenan were different. Furthermore, this demonstrates how even while operating under the identical conditions, the hydrogel’s dynamic diffusional process was distinct. The difference of the diffusion rate in the aqueous solution directly led to variation in the binding chance of TC426 in the film towards albumin in the solution. Hence, two crucial factors that govern whether TC426 and biomarkers continuously bind and persist in the film are the water diffusion rate (also referring to the albumin diffusion rate) and the polymer chain relaxation rate. In this case, the diffusion mechanism of the hydrogel system was studied according to Equations (2) and (3). The swelling power fractions versus time (Figure S8A,B) were plotted logarithmically to find the swell constant (K) and diffusional exponent (n). Diffusion information is summarised in Table 1. K was 0.012 (normal soaking) and 0.010 (VFD) in Aam–Alg, while n was 0.768 (normal soaking) and 0.917 (VFD). When the value of n was in the interval from 0.5 to 1, the kinetics of diffusion belonged to non-Fickian behaviours in AAm–Alg. In this situation, the swelling rate was greater than the rate of the film collapse, which means that the polymer relaxation was the rate-limiting step in the TC426–HSA delivery system. The relaxation rate in the hydrogel network became an uncontrollable factor, and the faster water penetration rate passively accelerated the relaxation progress, eventually leading to built-in TC426 leakage in the internal structures. In the carrageenan model, K was 0.142 (normal soaking) and 0.055 (VFD), while n was equal to 0.247 (normal soaking) and 0.403 (VFD). Less-Fickian (n < 0.45) was the diffusive feature of carrageenan as a diffusion-controlled delivery system and it dominated the albumin release process (the water penetration rate was much lower than the polymer chain relaxation rate). Equation (4) was also utilised to calculate the diffusion coefficient (D). The D value of AAm–Alg was 437.38-fold greater than that of carrageenan in the natural absorption, and 67.99 times greater under VFD rotation. The calculated diffusion coefficients D were consistent with the transport behaviour of the two hydrogel models. More importantly, the diffusion coefficient (D) of VFD processing was greater than that of normal soaking under the same model (2.85 times greater in AAm–Alg and 18.31 times greater in carrageenan). In other words, VFD had the effect of increasing the diffusion coefficient without the need for other external factors (temperature, concentrations, etc.).

Combined with the previous results, the template of non-Fickian diffusion was already at a high level of water penetration, and VFD further intensified it (D = 8950.06 × 10^−10^/m^2^ s^−1^). The bonding tightness between TC426 and the hydrogels is loosened at a high flow rate, thereby simultaneously increasing its fluorescent loss rate. However, the relaxation rate of the hydrogel chain was not significant in carrageenan due to Less-Fickian behaviour, and the diffusion coefficient (D = 131.63 × 10^−10^/m^2^ s^−1^, an order of magnitude lower than that of AAm–Alg) and dynamic loss rate (51.53% less than AAm–Alg) still had a relatively low and stable status, even under the action of VFD. The difference in loss rate also indicated a distinction in the internal structure of the two basements, and the lower loss percentage of carrageenan facilitated TC426 being locked inside.

#### 2.2.2. Microstructural Analysis

**Figure 3:**
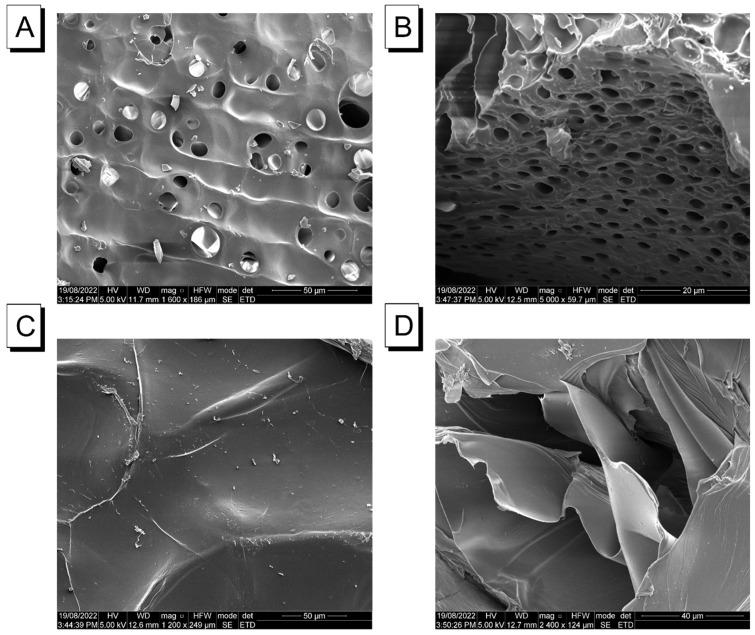
SEM images captured under 50 μm magnification from the surface of an (**A**) AAm–Alg + TC426 hydrogel film, (**C**) carrageenan + TC426 hydrogel film. SEM images captured under magnification 20 and 40 μm from the cross section of (**B**) AAm–Alg + TC426 hydrogel film, and (**D**) carrageenan + TC426 hydrogel film.

As previously indicated, the swelling behaviours, fluorescence kinetics, and diffusion of the two hydrogel matrices were different under the same conditions, and variation was likely linked with their microstructures. Therefore, scanning electron microscopy (SEM) was employed to obtain and explain the surface and cross-sectional topography of the dried samples at a high magnification. For the AAm–Alg-based hydrogels, the surface and cross section had a typical cellular porous structure, and the pore was relatively dense and evenly distributed (Figure 3A,B). The relatively loose network provided a sufficient adhering zone for the TC426 molecules and allowed for the aqueous solution of albumin to freely move within the film at a faster diffusion rate. A multilayer planar structure was characteristic of carrageenan on its surface and cross section (Figure 3C,D). Each layer exhibited a flat, smooth surface, but a certain gap existed layer to layer where the flow of water penetration was mainly singular and along the cross-sectional direction. More importantly, the structural morphology was an important factor determining the properties of the hydrogels. The porous space tended to diffuse in three dimensions, while multilayered structures were predominantly two-dimensional. This can be explained by the fact that, under the accelerated process of VFD, the AAm–Alg hydrogels could reach their maximal fluorescence intensity within 2 min while maintaining a constant higher swelling ratio compared to that of carrageenan. Likewise, TC426 could escape the constriction of the film along the porous network path as a result of the impact of the flow, resulting in higher dynamic loss. Furthermore, the layered structure of carrageenan resulted in a lower swelling ratio and allowed for it to readily restrict TC426 movement to maintain lesser loss while maintaining higher intensity.

After comprehending the microstructural differences of hydrogels, optical imaging with and without VFD-mediated circumstances could be further analysed. Figure S9A shows the case where only the cross linking of AAm–Alg was contained in the film. Its surface layer was flat and smooth. When macromolecular HSA was introduced into the model of Figure S9A, a black spherical substance with a white core was clearly visible on the hydrogel surface (Figure S9B). Dense granular microparticles could be easily captured in the same model containing only TC426 (Figure S9C). When both TC426 and albumin were present in the system, several bright spots appeared around the periphery of HSA, each represented by a black globular substance (Figure S9D). Similarly, in carrageenan, the blank sample exhibited a flat and smooth surface (Figure S10A), and a black spherical substance with a white core represented only HSA existance (Figure S10B). The AIE-embedded film also displayed dense granules (Figure S10C), and both TC426 and HSA could be independently observed in the coexisting system (Figure S10D).

**Figure 4:**
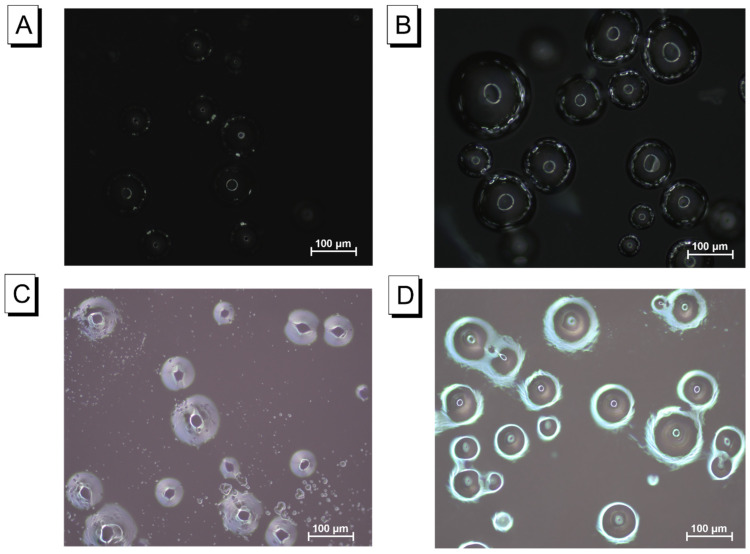
Optical microscopy images for the reflection mode in the darkfield. AAm–Alg hydrogel film + TC426: (**A**) without VFD; (**B**) with VFD, 100 μm; carrageenan hydrogel film + TC426: (**C**) without VFD; (**D**) with VFD, 100 μm; HSA = 2000 mg/L and TC426 = 10 μM, VFD was used for 2 and 4 min in AAm–Alg + TC426 and carrageenan + TC426, respectively.

In the darkfield, HSA spheres were covered with TC426 microparticles on the external edge that were then lit up (Figure 4A,C). Nevertheless, the strong green fluorescence was captured in the two matrices when VFD was applied during measurements (Figure 4B,D). The forming opportunities of the TC426–HSA aggregates increased as a result of the rise in the diffusion coefficient due to VFD shearing forces, which caused free albumin in the external solution to continuously be injected into the hydrogel interstitial space. Particularly, numerous TC426 connected with the binding sites on the albumin surface owing to dense contacting, thus increasing the steric hindrance. The white circle parts coating on albumin spheres represented the enrichments of aggregation state.

#### 2.2.3. Motion Analysis

**Figure 5:**
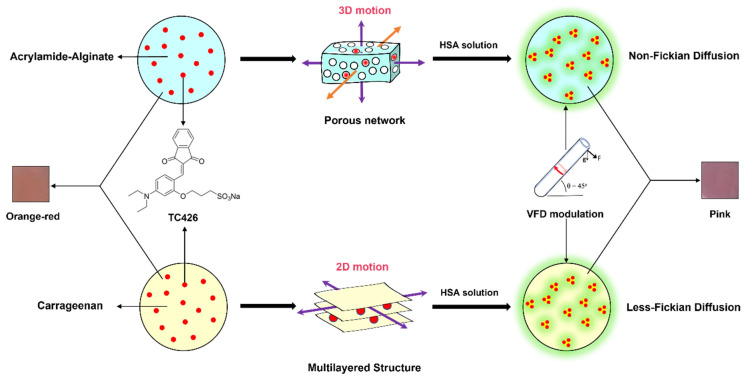
Potential mechanism of TC426-embedded AAm–Alg and carrageenan hydrogel film.

The underlying mechanism (Figure 5) of the hydrogels became clear after investigating their fluorescence kinetics, swelling behaviours, diffusion, and microstructure throughout the whole process. Initially, anionic sulfonate dye TC426 with AIE properties was bound to biological macromolecule HSA through noncovalent interactions, especially electrostatic interactions and hydrophobic effects. When TC426 molecules were docked onto the surface of the biomacromolecule and within the hydrophobic cavity of its folded structure, they clumped together by virtue of the strong electronic and hydrophobic interactions between their aromatic rings. Aggregation inhibited the intramolecular rotation of the TC426 molecules, thereby hindering their nonradiative transitions and activating their fluorescence emission processes. Subsequently, in the scenarios of hydrogel-based HSA detection with/without VFD, the porous network of AAm–Alg served as a fast-flow platform that enabled the albumin solution to simultaneously diffuse in multidimensional directions and further released the constraint of TC426 within the microstructure. Non-Fickian diffusion caused the relaxation rate of the AAm–Alg chain to become the delivery system’s rate-limiting step, which is difficult to be quantified in hydrogel models. Furthermore, the difference was that carrageenan’s layered structure provided only two-dimensional motion, while it affected diffusion to exhibit Less-Fickian behaviour, enabling the penetration rate of the HSA solution to dominate the transport process, and the passive diffusion-dominated delivery system to be regulated. More significantly, the introduction of VFD could artificially increase the diffusion rate under both models, and fluorescence intensity was greatly enhanced with the increased opportunity for a connection between HSA and TC426. By contrast, the diffusion-controlled delivery system of carrageenan was capable of boosting the diffusional rate while keeping dynamic losses constant. Without altering the original diffusion transport, the VFD acceleration procedure turned passive diffusion into active diffusion, which is a new control strategy for hydrogel studies.

### 2.3. Application of Portable Device Based on Colorimetry

**Figure 6:**
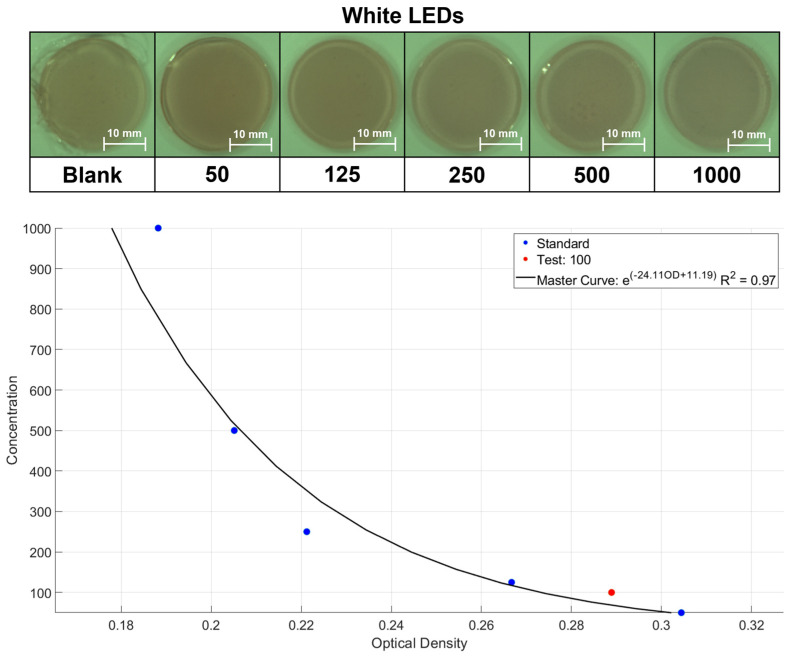
Correlations of HSA concentration and optical density in the range from 0 to 1000 mg/L under white LEDs. The black line represents the established master curve, R^2^ = 0.97; the red dot represents the validated sample. TC426 = 10 μM.

On the basis of our previously published studies [48,49,50], portable devices were developed for the quantitative detection of biomarkers. In this work, a colorimetric device (Figure S11) was developed for hydrogel films that utilises a three-layer RGB image matrix to output intensity values. The advantages of this approach are its sensitivity to colour shifting and being robust to different noise sources that may affect the image, such as sensor noise and artefacts. Since an obvious colour change was captured in carrageenan under the action of VFD, six spiked data points (5 blue dots and 1 blank control) were set to plot the standard curve. As shown in Figure 6, the red dot with 100 mg/L HSA concentration was input into the built-in arithmetic circle and it yielded a recovery rate of 86% optical density. The experiments demonstrated that this device could effectively identify the colour response of hydrogels towards HSA in the microalbumin range.

## 3. Experiments

### 3.1. Materials

#### 3.1.1. HSA and TC426

Albumin from human serum (A1653-5G) and dimethyl-sulfoxide (DMSO; 276855-2L) were purchased from Sigma-Aldrich, Australia, and bioprobe TC426 (Figure S1) was synthesised and characterised as previously reported [22].

#### 3.1.2. AAm–Alg–Ca^2+^ Hydrogel Film

For the PAAm/Alg–Ca^2+^ hydrogel, acrylamide (AAm; A8887) was the monomer used for the polyacrylamide (PAAm) networks; sodium alginate (SA; 2033; Pronova up LVM BP-1710-18) was the ionically cross-linkable biopolymer with calcium sulphate dehydrate (CaSO_4_·2H_2_O; C3771) as the ionic crosslinker for alginate; ammonium persulfate (APS; A3678) was used as the photo/thermal initiator for polyacrylamide; N,N-methylenebisacrylamide (MBAA; 146072) was used as a crosslinker for polyacrylamide; N,N,N’,N’-tetramethyl-ethylenediamine (TEMED; T9281) was used as a crosslinking accelerator for polyacrylamide. All chemicals were purchased from Sigma-Aldrich, Australia unless otherwise stated, and they were of analytical grade and used without further purification. Solutions were prepared with deionised water unless otherwise noted.

#### 3.1.3. Carrageenan Hydrogel Film

Carrageenan (GGOG, E407) was purchased from Sigma-Aldrich, Australia, and kept at room temperature for daily use.

### 3.2. Characterisations

A magnetic stirrer (Thermo Fisher Scientific Inc., Melbourne, VIC, Australia) was employed during hydrogel fabrication. Deionised water was produced from the Milli-Q Water Purification System (Merck Millipore Inc., Burlington, MA, USA). Laboratory Ovens (Labec Pty Ltd., Sydney, NSW, Australia) were equipped for heating. Fluorescence spectra were obtained on a Cary Eclipse Fluorescence Spectrophotometer (Agilent Technologies Inc., Santa Clara, CA, USA). A scanning electron microscope (SEM, FEI F50, Hillsboro, OR, USA) was employed to observe the microstructure. Optical imaging was performed with an Axio Imager 2 Upright microscope (Zeiss Inc., Oberkochen, Germany). Data were plotted using Origin 2019b (OriginLab Corp. Northampton, MA, USA).

### 3.3. Methods

#### 3.3.1. Preparation of HSA and TC426 Solution

We first prepared 2000 mg/L of an HSA solution in DI water as the stock solution and stored it at 0–4 °C. Then, it was diluted stepwise to the required concentrations in the range of 0–2000 mg/L in specific experiments for daily use. In addition, TC426 was dissolved in a DMSO solution to 10 mM as the stock solution and kept at 20 °C in the chemical cabinet for long-term storage.

#### 3.3.2. Hydrogel Fabrication of AAm–Alg–TC426

PAAm/Alg–Ca^2+^ hydrogel was synthesised on the basis of previously reported protocols with modifications [51]. We dissolved 480 mg of AAm and 76.4 µL of CaSO_4_ slurry (0.75 M) in 1 mL of deionised water to prepare Solution A. Then, 80 mg of SA, 14.4 µL MBAA (2% w/v), 3.2 µL TEMED, and 90.4 µL APS (0.27 M) were dissolved in 3 mL of deionised water to prepare Solution B. Afterwards, 4 μL of the TC426 dye solution ([TC426] = 10 μM refers to working concentration) was added into Solution B. Under magnetic stirring, Solutions A and B were quickly mixed to obtain a uniform solution. The solution was then poured into a mould, sealed, and heated up to 80 °C for 20 min to achieve free-radical polymerisation. Afterwards, the mould had been taken out and left at room temperature overnight (24 h) before the hydrogel was removed from the mould.

#### 3.3.3. Hydrogel Fabrication of Carrageenan–TC426

First, 80 mg of carrageenan powder was dissolved in 4 mL of deionised water under magnetic stirring; then, 4 μL TC426 dye solution (TC426 = 10 μM refers to the actual working concentration) was added after powder had fully dissolved until the two were mixed well together. Similarly, the final solution had been poured into the mould and sealed at room temperature overnight (24 h) before the hydrogel was removed from the mould.

#### 3.3.4. VFD Operation

The film was placed at the bottom of the VFD tube against the wall of the tube. Then, 3–4 mL of the specific albumin solution was poured, ensuring that the film was completely submerged below the liquid surface (Figure 1B,C). Rotational time was accurately controlled with a stopwatch, and one film was manipulated in each rotation.

#### 3.3.5. Protocols for TC426 + AAm–Alg/Carrageenan Hydrogels

The film had a fixed diameter of 25 mm and height of 2 mm (Figure S2), and the volume was 2 mL. Three random locations at each film were selected and tested as parallel control groups in every test; the excitation wavelength of TC426 was set at 480 nm. The VFD parameters for this study were 1500 rpm rotational speed, 45◦ tilt angle, clockwise (CW) rotation (looking down the tube), and rotational time was adjusted according to the specific experiment.

#### 3.3.6. Kinetics of Hydrogel Swelling

The swelling ratio is one of the most important parameters to characterise the swelling behaviour of hydrogel samples. In this experiment, the swelling performance of two basements of AIE-embedded materials, AAm–Alg and carrageenan, with or without the influence of VFD, was evaluated. Samples were weighed every minute in a time span from 0 to 10 min in groups of three, and the results were averaged to simultaneously standardise the behaviour and reduce errors. Excess water on the sample surface was wiped off with absorbent paper before each measurement.

The real-time swelling ratio can be expressed with the following formula [52]:

(1) $$Swellingratio\left(\%\right)=\frac{M_{t}-M_{e}}{M_{e}}\times 100\%$$

where $M_{t}$ is the actual film weight at t minutes, and $M_{e}$ is the film weight when it is in equilibrium (kept ≥ 24 h).

#### 3.3.7. Fraction of Swelling Power

The diffusion mechanism of hydrogels in aqueous solution is the primary element to study their swelling kinetics. Water diffusion motions induce a hydrogel to swell when hydrogels come into contact with water, which participates in the migration of water into pre-existing or dynamically formed spaces between the hydrogel chains. The fraction of swelling power can be derived from Fick’s law [53]:

(2) $${Fraction}_{swellingpower}=\frac{M_{t}-M_{0}}{M_{0}}=Kt^{n}$$

where $M_{t}$ is the mass of the swollen state at time t, and $M_{0}$ is the mass of the dry state at time 0. t is the time, K is the swelling constant, and n is the diffusional exponent. Equation (2) was only applied to the <60% stage in its swelling curve.

#### 3.3.8. Transport Determination 

The solvent–polymer system is characterised by constants K and diffusional exponent n. The physical process of hydrogel film absorption or AIE release was defined, and Equation (2) could be further derived to obtain diffusional exponent (n) to decide which type of transport in hydrogels. Particularly, natural logarithms are applied on both sides of Equation (2):

(3) $$ln(\frac{M_{t}-M_{0}}{M_{0}})=ln(Kt^{n})\to \ln\left(F_{sp}\right)=ln\left(K\right)+n*ln(t)$$

Therefore, diffusional exponent (n) can be calculated from the slopes of the lines of ln($F_{sp}$)–ln(t) plots. For cylindrical films, Fickian behaviour is indicated by 0.45 < n < 0.5; non-Fickian diffusion is defined as 0.50 < n < 1.0; when n < 0.45, it is less-Fickian behaviour. Crucially, diffusion transport that is decided by n can be used to predict the relationship between the penetration rate of water and the relaxation rate of polymer chains in swollen systems [54]. The information is displayed in Table 2.

#### 3.3.9. Diffusion Coefficient

The diffusion coefficient (D) generally describes the amount of a certain substance that diffuses across a unit area in one standard time while being affected by a one-unit gradient [55]. On the basis of Equation (3), K and n were obtained from cylindrical models of AAm–Alg and carrageenan hydrogels; then, the diffusion coefficient was calculated as follows [56]:

(4) $$D=\pi r^{2}{\left(\frac{K}{4}\right)}^{1/n}$$

where r represents the film radius at the swollen state, K is the swelling constant, and n is the diffusional exponent.

#### 3.3.10. Dynamic Loss

Fluorescence intensity loss is dynamic loss in this context. Since intensity is linearly positively associated with the quantity of TC426–HSA binding, and TC426 biosensors all originate from a film, hydrogel film is the source of all fluorescent signals, and the ratio of the fluorescence intensity for currently remaining films to the external solution, recorded in the VFD tube, could be expressed to reflect the actual leakage of TC426 in the delivery system. The equation is as follows:

(5) $$DynamicLoss\left(\%\right)=\frac{I_{e}}{I_{f}+I_{e}}\times 100\%$$

where $I_{e}$ is the FL intensity of the external HSA solution at different VFD times, and $I_{f}$ is the FL intensity of the currently existing hydrogel film.

#### 3.3.11. Limit of Detection

The limit of detection (LOD) represents the minimal fluorescence response of TC426–HSA aggregates in the presence of HSA. In this case, the LOD can be calculated with the following formula:

(6) $$LOD=\frac{3\sigma }{k}$$

where σ is the standard deviation of the blank measurement, and k is the slope of standard curve.

## 4. Conclusions

In summary, two fluorescent TC426-embedded hydrogels, AAm–Alg and carrageenan, were investigated for HSA detection under 0–10 min of normal soaking and VFD modulation. The results indicated that the fluorescence responses of the two hydrogel matrices were enhanced by 5.27-fold and 1.71-fold, respectively, when VFD was involved. The detection sensitivity of the linear dynamic curves was increased by 1.75-fold and 2.56-fold, respectively, while the LOD was reduced to 61.19 and 30.87 mg/L. Through the explanation of the potential mechanism, the primary cause for the non-Fickian behaviour was the porous network of AAm–Alg, whose D was 3143.6 × 10^−10^/m^2^ s^−1^, and the multilayered structure of carrageenan led to Less-Fickian behaviour under the same circumstances, resulting in a smaller diffusion coefficient (7.187 × 10^−10^/m^2^ s^−1^). Significantly, VFD could speed up the diffusion rate without changing its initial transport mode (2.85-fold for AAm–Alg and 18.31-fold for carrageenan). After that, motion analysis showed that the movement of the HSA solution in three- or two-dimensional directions under the hydrogel microstructure was the rate-limiting factor that determined the transport system. The diffusion-dominated system led by carrageenan was more capable of increasing fluorescence intensity while maintaining the dynamic loss under VFD control. Furthermore, a recovery rate of 86% from the colorimetric device successfully validated that the colour change in the fluorescent hydrogels could be recognised in the microalbumin range after VFD processing. VFD could convert passive into active diffusion, which is a promising strategy for future hydrogel research.

## Figures and Tables

**Table 1:** Diffusional exponent (n), type of transport, actual behaviours, and diffusion coefficient (D) in normal soaking and VFD testing for hydrogel films of AAm–Alg and carrageenan.

Hydrogel Material	Testing Type	Diffusional Exponent (n)	Type of Transport	Behaviour	Diffusion Coefficient (D × 10^−10^/m^2^ s^−1^)
AAm–Alg	Normal soaking	0.768	Non-Fickian diffusion	R_water penetration_ > R_polymer chain relaxation_	3143.6
	VFD	0.917			8950.1
Carrageenan	Normal soaking	0.247	Less-Fickian diffusion	R_water penetration_ ≪ R_polymer chain relaxation_	7.187
	VFD	0.403			131.632

**Table 2:** Definition of three types of diffusion transport and their corresponding factors.

Transport	Diffusional Exponent (n)	Actual Behaviours
Less-Fickian	n < 0.45	R_water penetration_ ≪ R_polymer chain relaxation_
Fickian	0.45 < n < 0.5	R_water penetration_ < R_polymer chain relaxation_
Non-Fickian	0.50 < n < 1.0	R_water penetration_ > R_polymer chain relaxation_

## Data Availability

Data supporting reported results can be obtained as requested.

## Supplementary Materials

The following are available online at https://www.mdpi.com/article/10.3390/molecules28073244/s1, Figure S1. 1H NMR in DMSO for TC426, Figure S2. The mould of hydrogel film, Figure S3. Hydrogel film with embedded AIE biosensor TC426 reacting with different concentrations of HSA solution, Figure S4. Acrylamide + Alginate + TC426 Hydrogel film for real observation, Figure S5. Carrageenan + TC426 Hydrogel film for real observation, Figure S6. Hydrogel film for normal soaking testing and VFD processing testing, Figure S7. Comparison between AAm + Alg + TC426 film and Carrageenan + TC426 film, Figure S8. Change in the fraction of swelling power (Fsp) vs. time, Figure S9. The images of optical microscopy in reflected light, Figure S10. The images of optical microscopy in reflected light, Figure S11. The 3D structure of Portable Colorimetric Device for optical imaging analysis.
