# Supplementary material for: Hydrogel-Film-Fabricated Fluorescent Biosensors with Aggregation-Induced Emission for Albumin Detection through the Real-Time Modulation of a Vortex Fluidic Device
Source: Molecules. 2023 Apr 5;28(7):3244. doi: 10.3390/molecules28073244 (PMC10096627; doi:10.3390/molecules28073244)
Supplement: Supplementary file 1 [file molecules-28-03244-s001.zip › molecules-2306709-supplementary.pdf]

Supporting information

Hydrogel-Film-Fabricated Fluorescent Biosensors with Aggregation-Induced Emission for Albumin Detection through the Real-Time Modulation of a Vortex Fluidic Device

Qi Hu [1,2], Xuan Luo [2], Damian Tohl [1], Anh Tran Tam Pham [1], Colin Raston [2] and Youhong Tang [1,2,*]

[1] Australia-China Joint Research Centre on Personal Health Technologies, Medical Device Research Institute, Flinders University, Adelaide, SA 5042, Australia

[2] Institute for NanoScale Science and Technology, College of Science and Engineering, Flinders University, Adelaide, SA 5042, Australia

* Correspondence: youhong.tang@flinders.edu.au; Tel.: +61-8-82012138

Table of Figures

Figure S1. ^{1}H NMR in DMSO for TC426. 4

Figure S2. The mould of hydrogel film. (A) Template of flat design illustration and the diameter is 25 mm; (B) Template of spatial design illustration and thickness is 2 mm. 5

Figure S3. Hydrogel film with embedded AIE biosensor **TC426** reacting with different concentrations of HSA solution. Fluorescence spectra of (A) **AAm-Alg** + **TC426** and (B) **Carrageenan** + **TC426** in the presence of HSA in the range of 0 to 1000 mg/L; [**TC426**] = 10 μM, λ_{ex} = 480 nm. 6

Figure S4. **Acrylamide** + **Alginate** + **TC426** Hydrogel film for real observation. (A) Monitoring of colour change and swelling change from 0 to 10 min during normal soaking testing; (B) Monitoring of colour change and swelling change from 0 to 10 min during VFD testing. [**TC426**] = 10 μM, [HSA] = 2000 mg/L, λ_{ex} = 480 nm, I_0 equals the intensity of [HSA] = 0 mg/L. 7

Figure S5. **Carrageenan** + **TC426** Hydrogel film for real observation. (A) Monitoring of colour change and swelling change from 0 to 10 min during normal soaking testing; (B) Monitoring of colour change and swelling change from 0 to 10 min during VFD testing. [**TC426**] = 10 μM, [HSA] = 2000 mg/L, λ_{ex} = 480 nm, I_0 equals the intensity of [HSA] = 0 mg/L. 8

Figure S6. Hydrogel film for normal soaking testing and VFD processing testing. (A) **Acrylamide** + **Alginate** + **TC426** and (C) **Carrageenan** + **TC426** for time effect on variation of intensity and swelling ratio from 0 to 10 min during normal soaking testing; (B) **Acrylamide** + **Alginate** + **TC426** and (D) **Carrageenan** + **TC426** for time effect on variation of intensity and swelling ratio from 0 to 10 min during VFD testing. [**TC426**] = 10 μM, [HSA] = 2000 mg/L, λ_{ex} = 480 nm, I_0 equals the intensity of [HSA] = 0 mg/L. 9

Figure S7. Comparison between **AAm** + **Alg** + **TC426** film and **Carrageenan** + **TC426** film. (A) Loss ratio from 0 to 10 min during VFD testing. (B) Time effect when VFD processing is

immediately completed and 3 hours after the completion. [**TC426**] = 10 μM, [HSA] = 2000 mg/L, λ_{ex} = 480 nm, VFD was conducted for 2 minutes and 4 minutes, towards **AAm** + **Alg** + **TC426** and **Carrageenan** + **TC426** respectively..........10

Figure S8. Change in the fraction of swelling power (F_{sp}) vs. time to identify the diffusion mechanism in normal soaking and VFD tests of (A) **Carrageenan** + **TC426** film and (B) **AAm** + **Alg** + **TC426** film..........11

Figure S9. The images of optical microscopy in reflected light. (A) **AAm** + **Alg** hydrogel film only, 100 μm; (B) **AAm** + **Alg** hydrogel film + HSA, [HSA] = 2000 mg/L, 100 μm; (C) **AAm** + **Alg** hydrogel film + **TC426**, [TC426] = 10μM, 100 μm; (D) **AAm** + **Alg** hydrogel film + HSA + **TC426**, [HSA] = 2000 mg/L and [**TC426**] = 10μM, 100 μm..........12

Figure S10. The images of optical microscopy in reflected light. (A) **Carrageenan** hydrogel film only, 100 μm; (B) **Carrageenan** hydrogel film + HSA, [HSA] = 2000 mg/L, 200 μm; (C) **Carrageenan** hydrogel film + **TC426**, [**TC426**] = 10μM, 100 μm; (D) **Carrageenan** hydrogel film + HSA + **TC426**, [HSA] = 2000 mg/L and [**TC426**] = 10μM, 100 μm..........13

Figure S11. The 3D structure of Portable Colorimetric Device for optical imaging analysis; Components illustration including camera group, environmental light-proof case, base, 4 LED modules and 4 reference points..........14

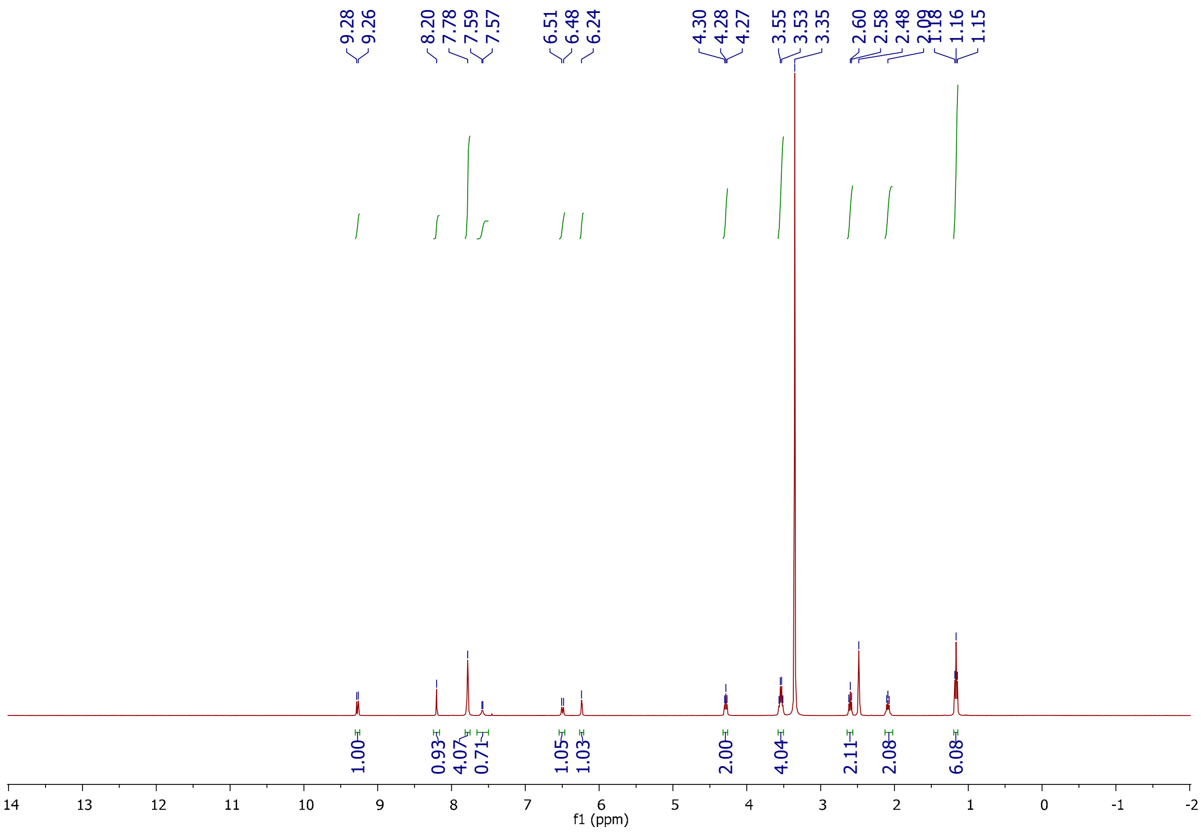

Figure S1. ^{1}H NMR in DMSO for TC426 [s1].

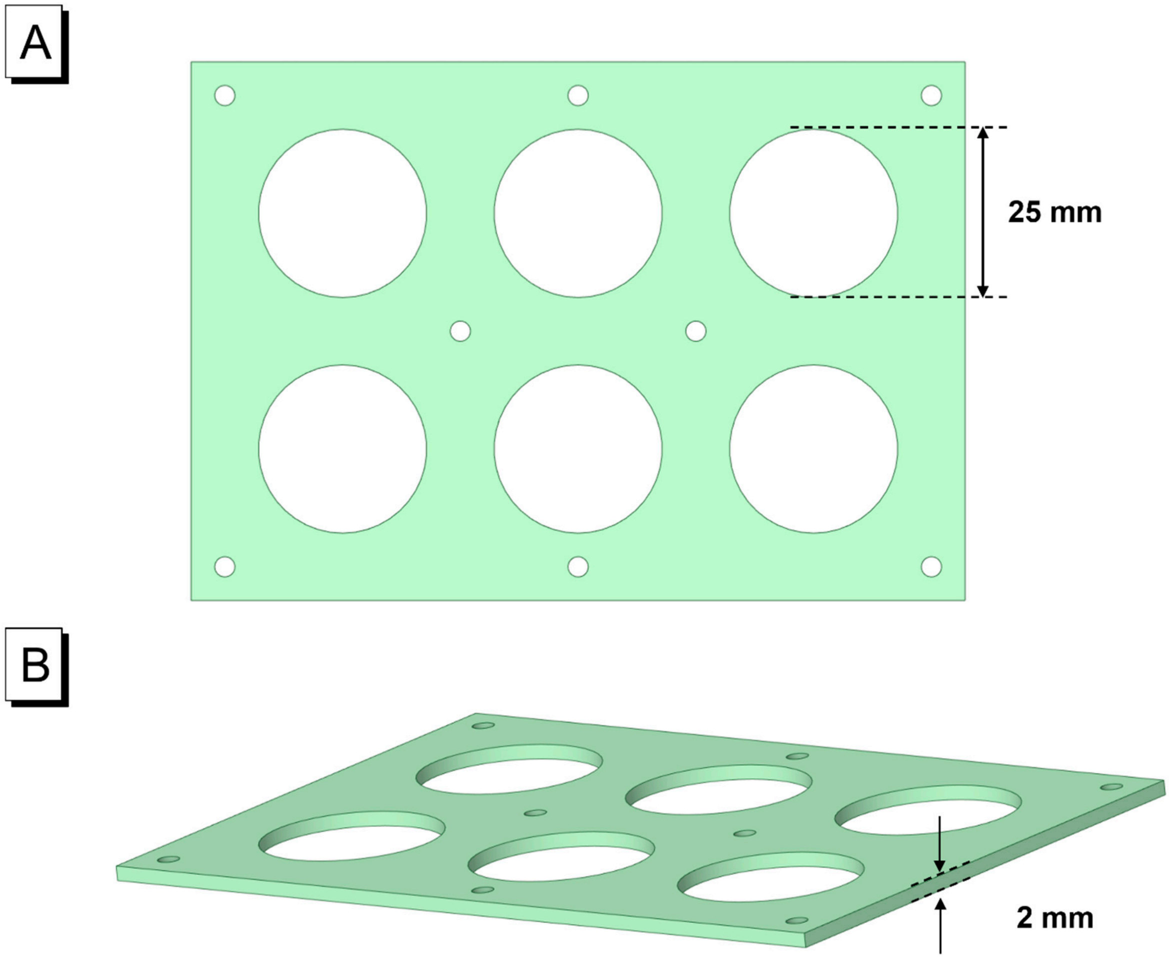

Figure S2. The mould of hydrogel film. (A) Template of flat design illustration and the diameter is 25 mm; (B) Template of spatial design illustration and thickness is 2 mm.

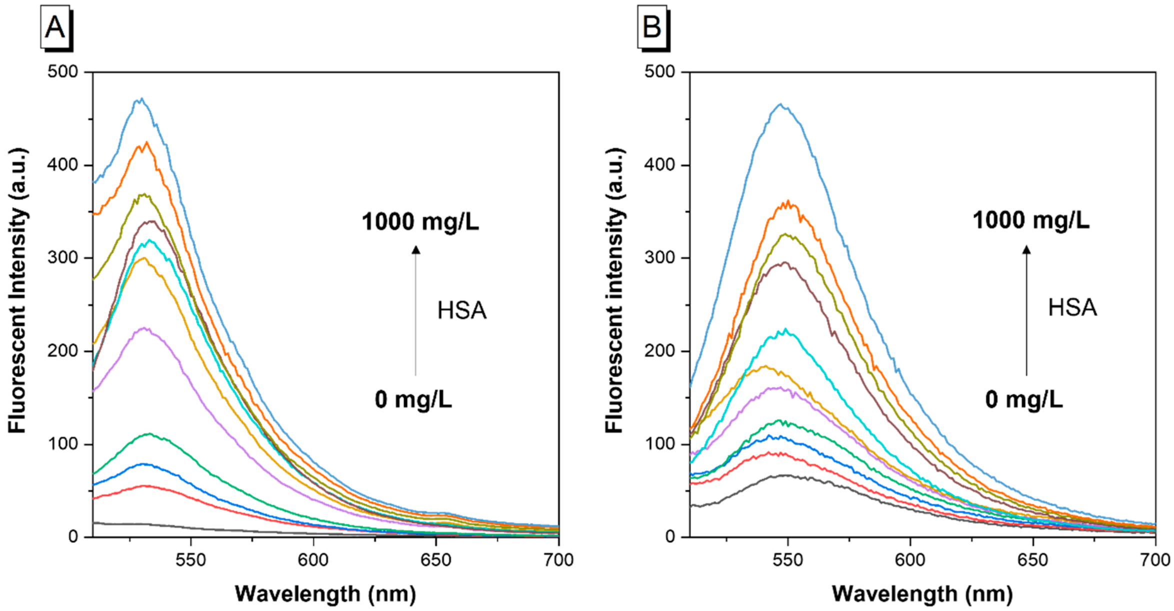

Figure S3. Hydrogel film with embedded AIE biosensor **TC426** reacting with different concentrations of HSA solution. Fluorescence spectra of (A) **AAm-Alg** + **TC426** and (B) **Carrageenan** + **TC426** in the presence of HSA in the range of 0 to 1000 mg/L; [**TC426**] = 10 μM, λ_{ex} = 480 nm.

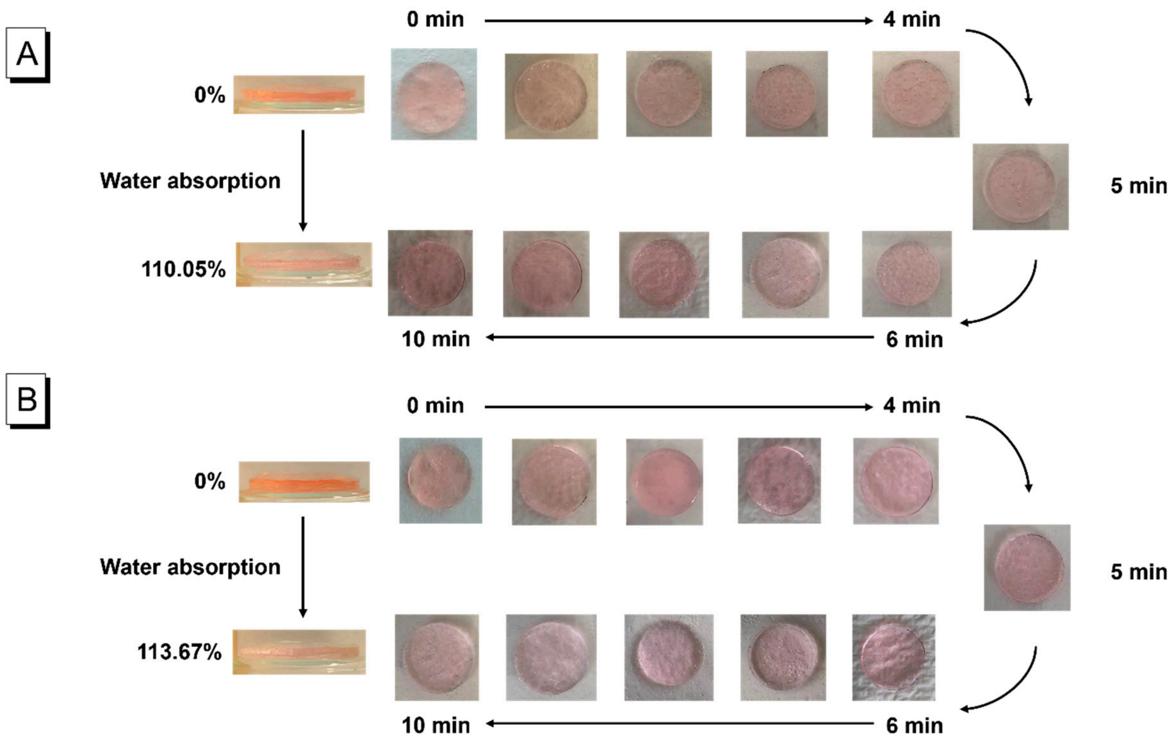

Figure S4. **Acrylamide** + **Alginate** + **TC426** Hydrogel film for real observation. (A) Monitoring of colour change and swelling change from 0 to 10 min during normal soaking testing; (B) Monitoring of colour change and swelling change from 0 to 10 min during VFD testing. [**TC426**] = 10 μM, [HSA] = 2000 mg/L, λ_{ex} = 480 nm, I_0 equals the intensity of [HSA] = 0 mg/L.

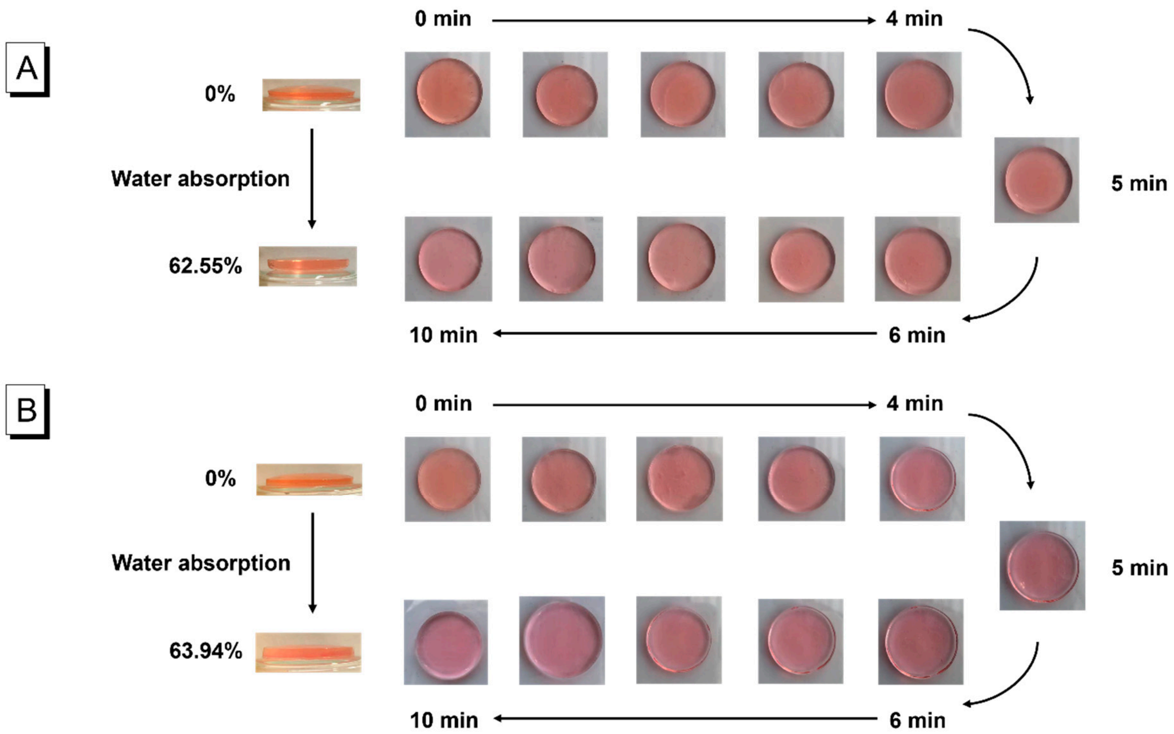

Figure S5. **Carrageenan** + **TC426** Hydrogel film for real observation. (A) Monitoring of colour change and swelling change from 0 to 10 min during normal soaking testing; (B) Monitoring of colour change and swelling change from 0 to 10 min during VFD testing. [**TC426**] = 10 µM, [HSA] = 2000 mg/L, λ_{ex} = 480 nm, I_0 equals the intensity of [HSA] = 0 mg/L.

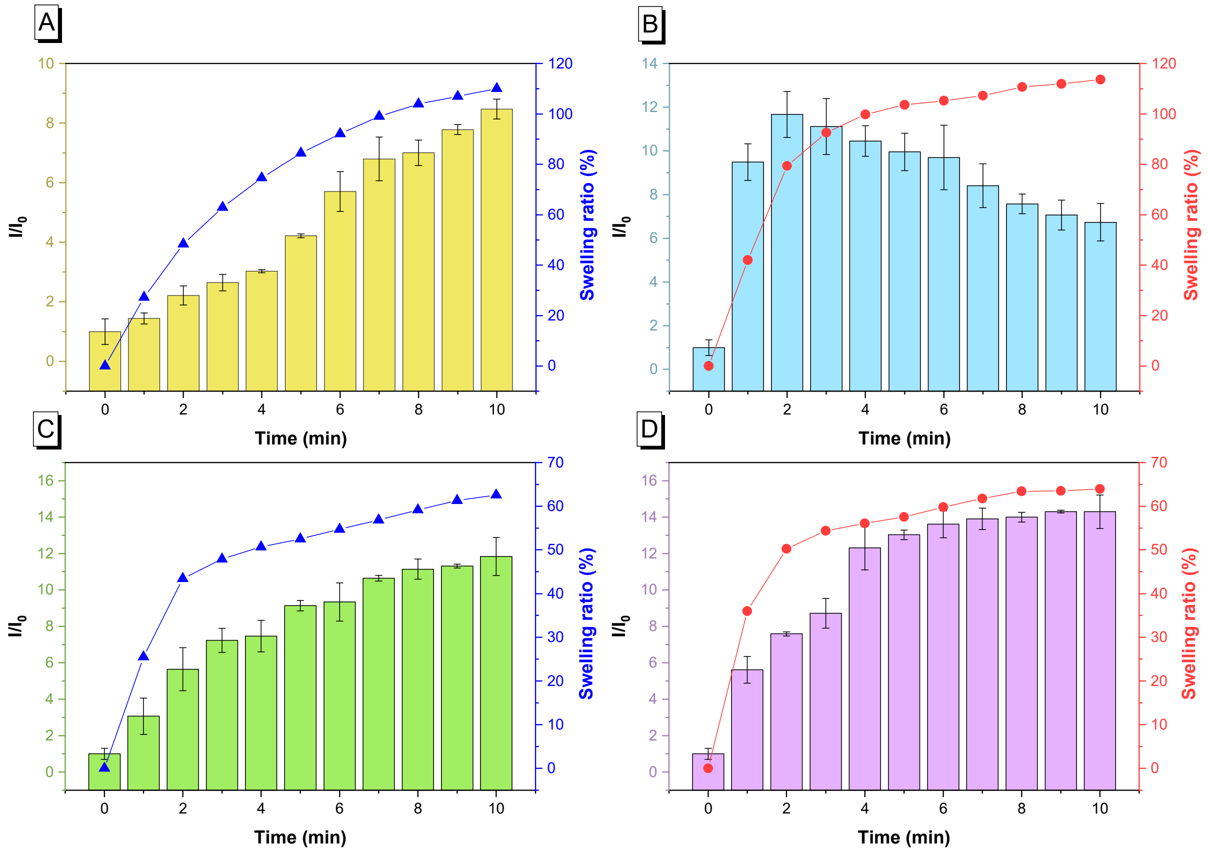

Figure S6. Hydrogel film for normal soaking testing and VFD processing testing. (A) **Acrylamide** + **Alginate** + **TC426** and (C) **Carrageenan** + **TC426** for time effect on variation of intensity and swelling ratio from 0 to 10 min during normal soaking testing; (B) **Acrylamide** + **Alginate** + **TC426** and (D) **Carrageenan** + **TC426** for time effect on variation of intensity and swelling ratio from 0 to 10 min during VFD testing. [**TC426**] = 10 µM, [HSA] = 2000 mg/L, λ_{ex} = 480 nm, I_0 equals the intensity of [HSA] = 0 mg/L.

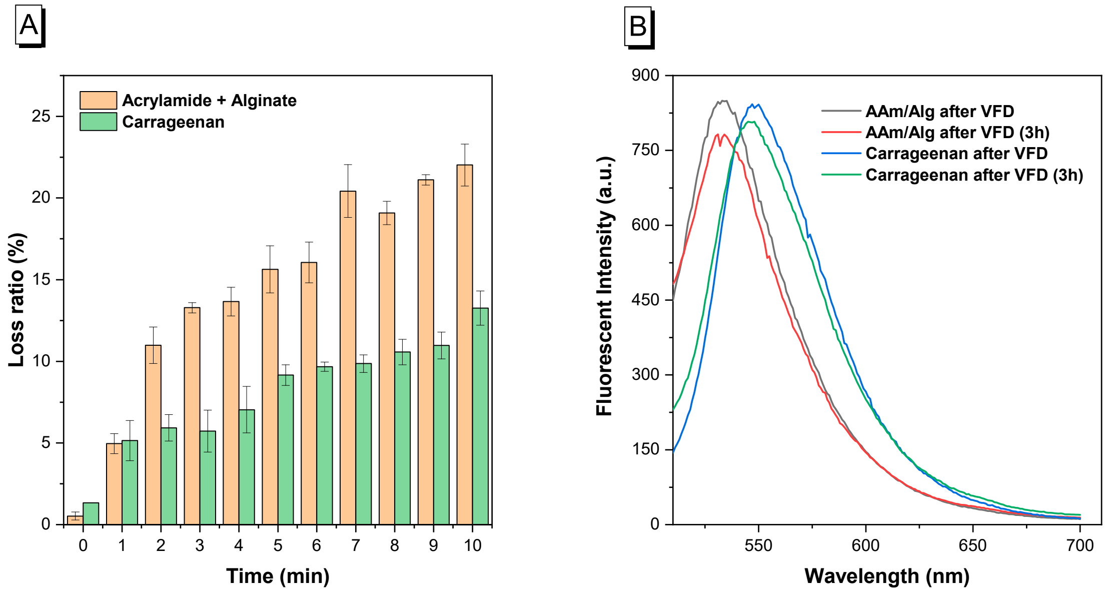

Figure S7. Comparison between **AAm** + **Alg** + **TC426** film and **Carrageenan** + **TC426** film. (A) Loss ratio from 0 to 10 min during VFD testing. (B) Time effect when VFD processing is immediately completed and 3 hours after the completion. [**TC426**] = 10 μM, [HSA] = 2000 mg/L, λ_{ex} = 480 nm, VFD was conducted for 2 minutes and 4 minutes, towards **AAm** + **Alg** + **TC426** and **Carrageenan** + **TC426** respectively.

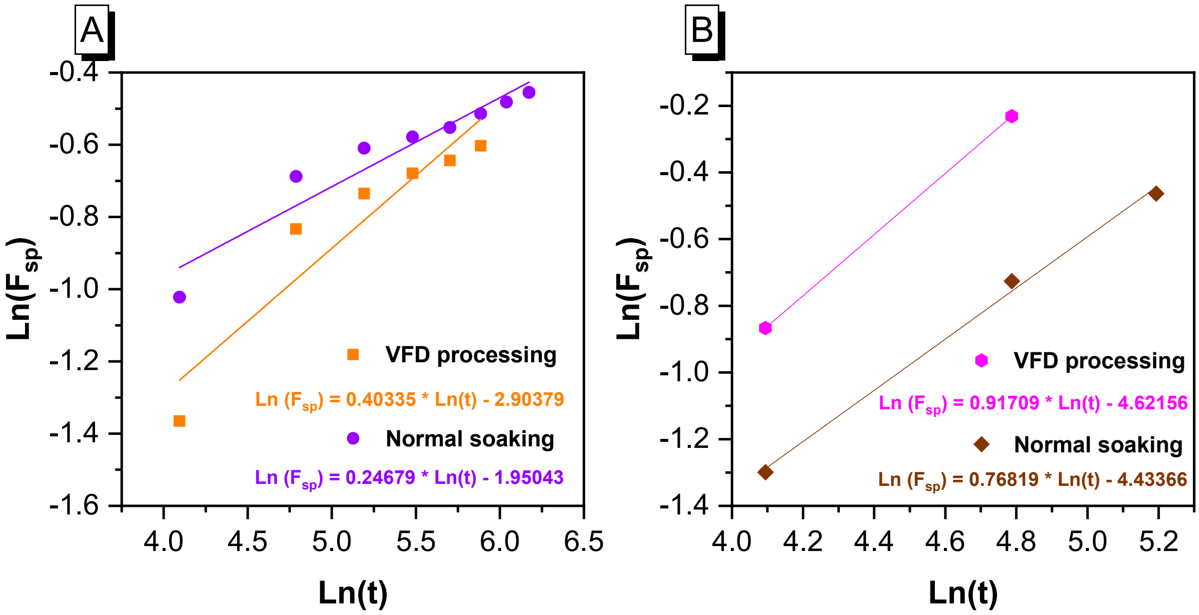

Figure S8. Change in the fraction of swelling power (F_{sp}) vs. time to identify the diffusion mechanism in normal soaking and VFD tests of (A) **Carrageenan** + **TC426** film and (B) **AAm** + **Alg** + **TC426** film.

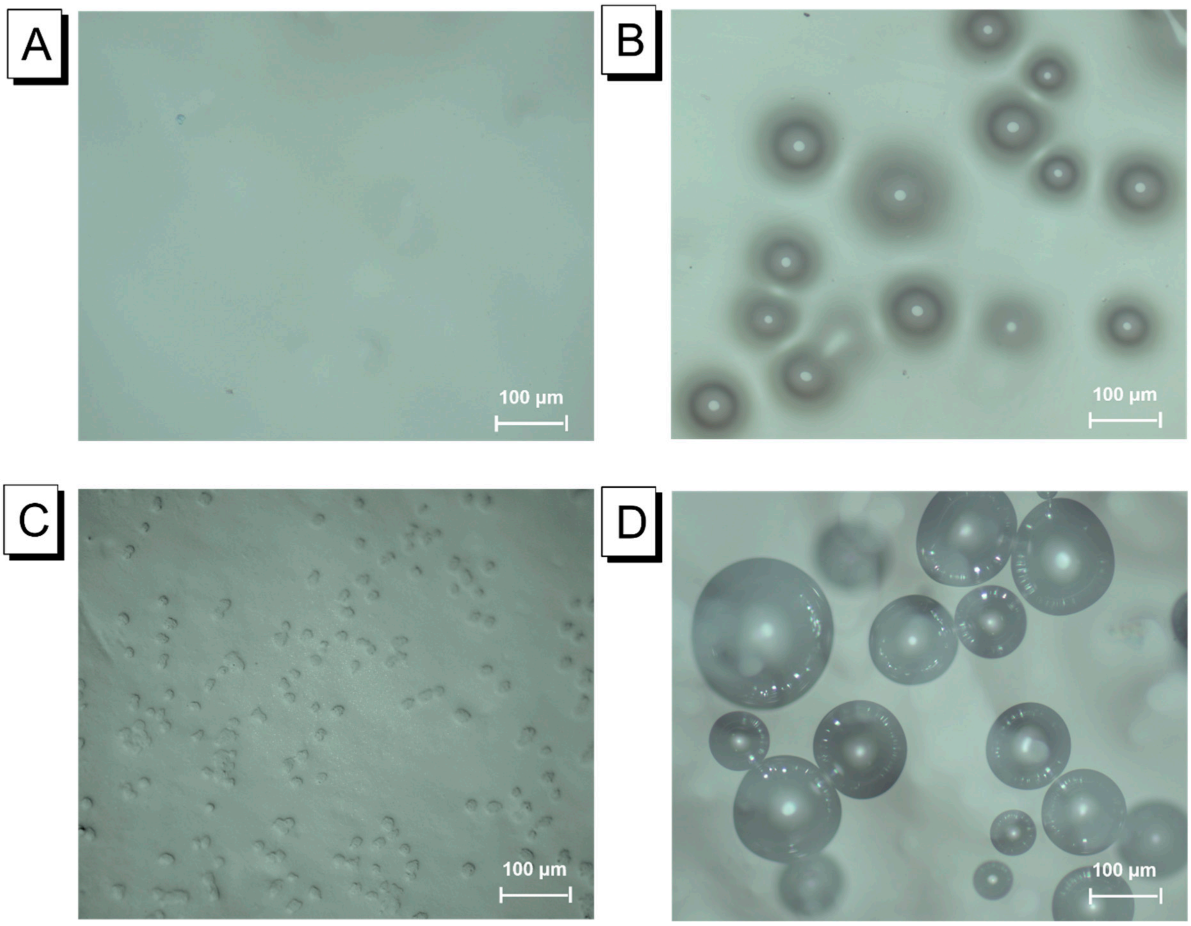

Figure S9. The images of optical microscopy in reflected light. (A) **AAm** + **Alg** hydrogel film only, 100 µm; (B) **AAm** + **Alg** hydrogel film + HSA, [HSA] = 2000 mg/L, 100 µm; (C) **AAm** + **Alg** hydrogel film + **TC426**, [TC426] = 10µM, 100 µm; (D) **AAm** + **Alg** hydrogel film + HSA + **TC426**, [HSA] = 2000 mg/L and [**TC426**] = 10µM, 100 µm.

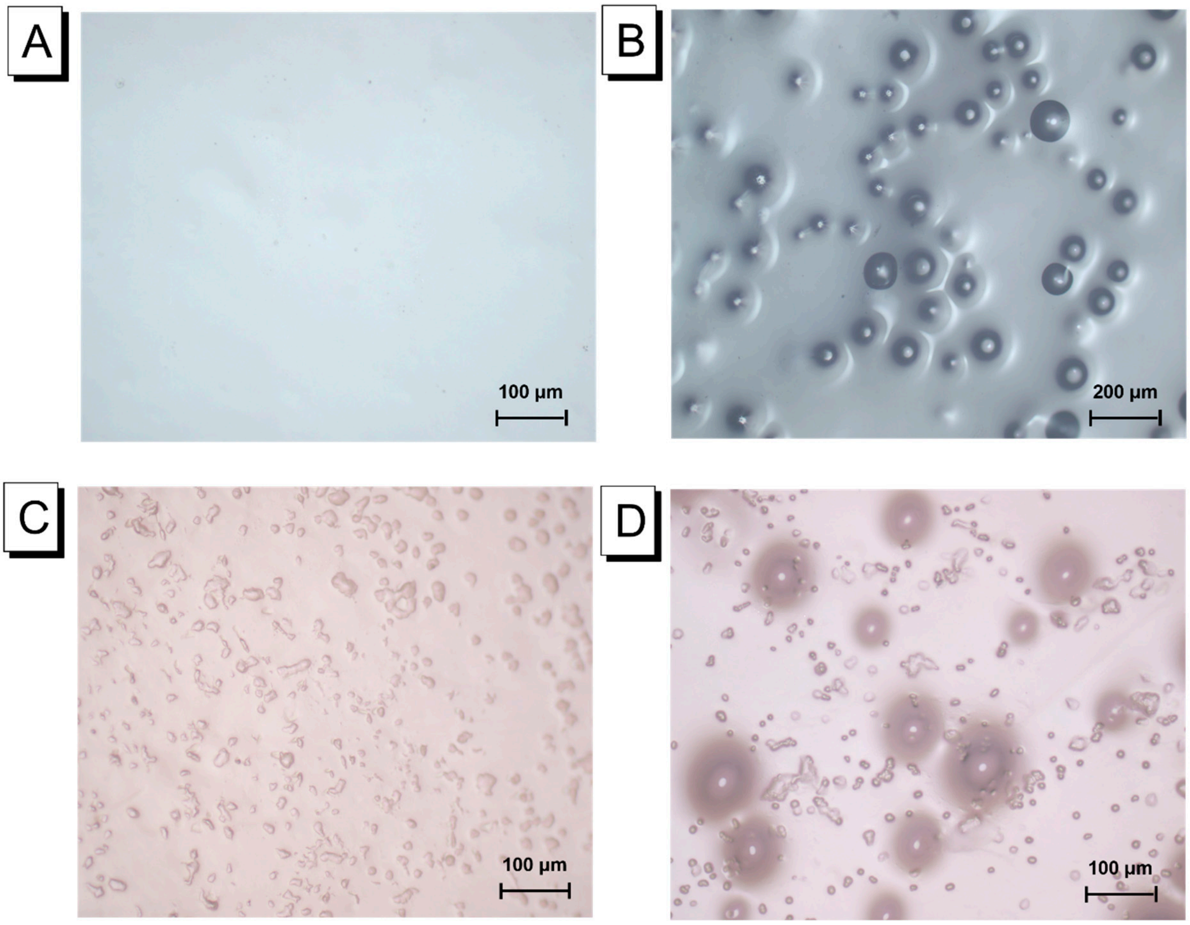

Figure S10. The images of optical microscopy in reflected light. (A) **Carrageenan** hydrogel film only, 100 µm; (B) **Carrageenan** hydrogel film + HSA, [HSA] = 2000 mg/L, 200 µm; (C) **Carrageenan** hydrogel film + **TC426**, [**TC426**] = 10µM, 100 µm; (D) **Carrageenan** hydrogel film + HSA + **TC426**, [HSA] = 2000 mg/L and [**TC426**] = 10µM, 100 µm.

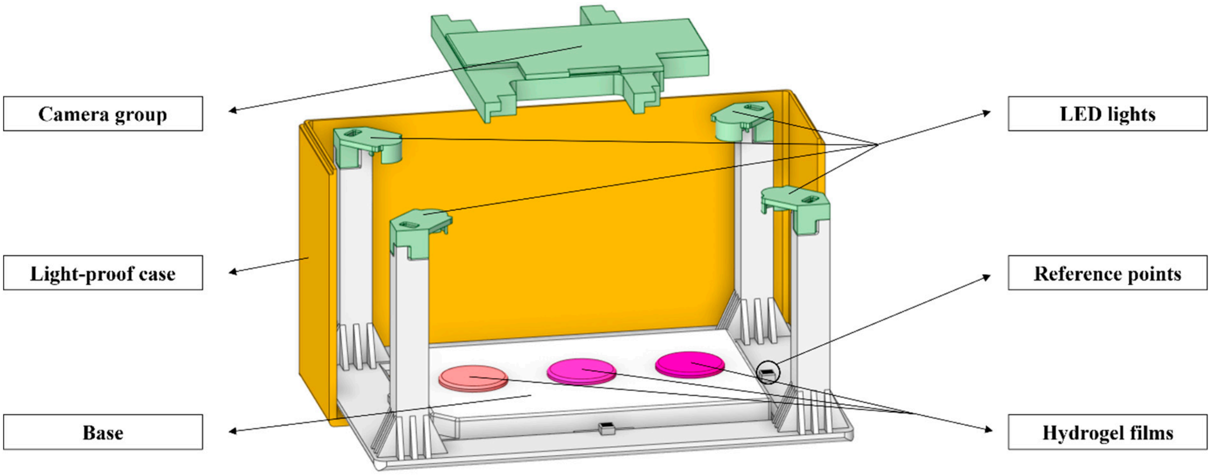

Figure S11. The 3D structure of portable colorimetric device for optical imaging analysis; Components illustration including camera group, environmental light-proof case, base, 4 LED modules and 4 reference points (Modified with [s2]).
